# Noninsulin Antidiabetic Drugs for Patients with Type 2 Diabetes Mellitus: Are We Respecting Their Contraindications?

**DOI:** 10.1155/2016/7502489

**Published:** 2016-01-06

**Authors:** Irene Ruiz-Tamayo, Josep Franch-Nadal, Manel Mata-Cases, Dídac Mauricio, Xavier Cos, Antonio Rodriguez-Poncelas, Joan Barrot, Gabriel Coll-de-Tuero, Xavier Mundet-Tudurí

**Affiliations:** ^1^Primary Health Care Center La Torrassa, Consorci Sanitari Integral, Ronda Torrassa 151-153, 08903 L'Hospitalet de Llobregat, Spain; ^2^DAP-Cat Group, Unitat de Suport a la Recerca Barcelona Ciutat, Institut Universitari d'Investigació en Atenció Primària Jordi Gol (IDIAP Jordi Gol), Sardenya 375, 08006 Barcelona, Spain; ^3^Primary Health Care Center Raval Sud, Gerència d'Àmbit d'Atenció Primària Barcelona Ciutat, Institut Català de la Salut, Avinguda Drassanes 17-21, 08001 Barcelona, Spain; ^4^CIBER of Diabetes and Associated Metabolic Diseases (CIBERDEM), Instituto de Salud Carlos III (ISCIII), Monforte de Lemos 3-5, 28029 Madrid, Spain; ^5^Primary Health Care Center La Mina, Gerència d'Àmbit d'Atenció Primària Barcelona Ciutat, Institut Català de la Salut, Mar S/N, 08930 Sant Adrià de Besòs, Spain; ^6^Department of Endocrinology & Nutrition, Health Sciences Research Institute and Hospital Universitari Germans Trias i Pujol, Carretera Canyet S/N, 08916 Badalona, Spain; ^7^Primary Health Care Center Sant Martí de Provençals, Gerència d'Àmbit d'Atenció Primària Barcelona Ciutat, Institut Català de la Salut, Fluvià 211, 08020 Barcelona, Spain; ^8^Primary Health Care Center Anglès, Gerència d'Àmbit d'Atenció Primària Girona, Institut Català de la Salut, Carretera de Girona S/N, 17160 Anglès, Spain; ^9^Primary Health Care Center Salt, Gerència d'Àmbit d'Atenció Primària Girona, Institut Català de la Salut, Manel de Falla 35, 17190 Salt, Spain; ^10^Primary Health Care Center El Carmel, Gerència d'Àmbit d'Atenció Primària Barcelona Ciutat, Institut Català de la Salut, Murtra 130, 08032 Barcelona, Spain; ^11^Autonomous University of Barcelona, Campus de Bellaterra, 08193 Bellaterra, Spain

## Abstract

*Aim*. To assess prescribing practices of noninsulin antidiabetic drugs (NIADs) in T2DM with several major contraindications according to prescribing information or clinical guidelines: renal failure, heart failure, liver dysfunction, or history of bladder cancer.* Methods*. Cross-sectional, descriptive, multicenter study. Electronic medical records were retrieved from all T2DM subjects who attended primary care centers pertaining to the Catalan Health Institute in Catalonia in 2013 and were pharmacologically treated with any NIAD alone or in combination.* Results*. Records were retrieved from a total of 255,499 pharmacologically treated patients. 78% of patients with some degree of renal impairment (glomerular filtration rate (GFR) < 60 mL/min) were treated with metformin and 31.2% with sulfonylureas. Even in the event of severe renal failure (GFR < 30 mL/min), 35.3% and 22.5% of patients were on metformin or sulfonylureas, respectively. Moreover, metformin was prescribed to more than 60% of patients with moderate or severe heart failure.* Conclusion*. Some NIADs, and in particular metformin, were frequently used in patients at high risk of complications when they were contraindicated. There is a need to increase awareness of potential inappropriate prescribing and to monitor the quality of prescribing patterns in order to help physicians and policymakers to yield better clinical outcomes in T2DM.

## 1. Introduction

Lifestyle modification, primarily through diet, and exercise advise are the preferred therapeutic approaches in the initial treatment of type 2 diabetes mellitus (T2DM). However, the disease tends to progress and most patients will be required to start on oral medication to maintain individualized glycemic targets. Noninsulin antidiabetic drugs (NIADs) are typically the first option for initial pharmacotherapy and include different classes of drugs with diverse modes of action, therapeutic potency, and adverse reactions [1].

Some NIADs have contraindications or must be used with caution in patients with T2DM and particular comorbid conditions. Major at-risk conditions that require tailored management of hyperglycemia include heart failure, chronic kidney disease, liver dysfunction, or history of bladder cancer. Moderate to severe renal impairment, for instance, is present in 20–40% of T2DM patients [2–4], which requires careful evaluation of risks and benefits when prescribing antihyperglycemic drugs with renal clearance. In addition, the prevalence of chronic heart failure in T2DM is 10–23%, and they have 2-fold greater risk of heart failure than their nondiabetic counterparts [5]. Suboptimal glycemic control is a predictor for its development [6]; furthermore, some glucose-lowering agents may be associated with an increased risk of heart failure [7]. Finally, T2DM patients also have an increased prevalence of the entire spectrum of liver disease, from abnormal liver enzyme levels to acute liver failure, and the use of particular antihyperglycemic agents must be avoided or they must be used with caution due to altered drug metabolism and/or hepatotoxicity [8].

Besides prescribing information enclosed in the package insert or the summary of product characteristics (SmPC), which list the labels and contraindications of each particular drug, local health authorities, international expert consensus documents, and clinical guidelines regularly publish recommendations on the use of antidiabetic drugs and indicate in which comorbid conditions they are formally contraindicated [9–12].

The appropriate use of NIADs in accordance with prescribing information or recommendations is of great importance to preserve or increase quality of life, particularly in patients with comorbid disease conditions [13]. However, a poor adherence to local and/or international guidelines on T2DM management has been reported in both primary and secondary care settings, and inappropriate or potentially inappropriate prescription of NIADs to patients with a contraindication or precautionary condition has also been documented [14–19].

The aim of this study was to investigate prescribing patterns of NIADs in a primary care setting in Catalonia, Spain, in patients with some at-risk comorbid conditions and assessed whether treatment choices agreed with the current drug's prescribing information, recommendations of expert consensus documents, or clinical guidelines when they are formally contraindicated or recommended to be used with caution.

## 2. Methods

### 2.1. Design

This was a cross-sectional, descriptive, multicenter study including all type 2 diabetes subjects between 31 and 90 years of age who attended any of the 274 primary care centers pertaining to the Catalan Health Institute (ICS) in Catalonia, Spain, in 2013. Electronic medical records were retrieved from the SIDIAP database (System for the Development of Research in Primary Care) as previously reported [4, 20]. Subjects were included in the study if they had a T2DM diagnosis (ICD-10 codes E11, E11.0–E11.9, E14, or E14.0–E14.9) in the electronic clinical record and were prescribed pharmacological treatment with any NIAD in monotherapy or in combination. Those patients exclusively treated with lifestyle modification or insulin as monotherapy were excluded.

### 2.2. Studied Variables

The study included data on age; gender; duration of T2DM; standardized glycated hemoglobin (HbA1c) values, using the most recent value of the preceding 15 months; and risk factors and diabetic complications, including body mass index (BMI) (most recent value in the last 24 months), microvascular complications (diabetic retinopathy, nephropathy or neuropathy), and macrovascular complications (coronary artery disease, recent myocardial infarction of a duration less than 1 year, stroke, peripheral artery disease, and heart failure). Pharmacological treatments were extracted from prescription- and pharmacy-invoicing data provided by the CatSalut general database and included the use of any NIADs as monotherapy or in combination with other glucose-lowering drugs (e.g., insulin) licensed at that time in Spain, namely, metformin, sulfonylureas, meglitinides, alpha-glucosidase inhibitors (AGIs), pioglitazone, dipeptidyl peptidase-4 inhibitors (DPP-4i), and glucagon-like peptide-1 receptor agonists (GLP-1ra).

The following major conditions were considered a potential contraindication for some drugs based on the summary of product characteristics (SmPC), international expert consensus documents, or clinical guidelines: (i) renal failure, defined as a glomerular filtration rate (GFR) < 60 mL/min/1.73 m^2^, and severe renal failure (GFR < 30 mL/min/1.73 m^2^), estimated with the Chronic Kidney Disease Epidemiology Collaboration (CKD-EPI) equation [21]; (ii) liver dysfunction, defined as hepatic enzymes over 3 times the upper limit of normal levels (either glutamyl oxaloacetic transaminase (GOT) or glutamyl pyruvic transaminase (GPT) > 120 IU/L or gamma-glutamyl transferase (GGT) > 150 IU/L); (iii) heart failure (globally and New York Heart Association (NYHA) class III or IV) functional stage [22]; and (iv) history of bladder cancer.

This study was approved by the Ethics Committee of the Primary Health Care University Research Institute (IDIAP) Jordi Gol.

### 2.3. Statistical Analysis

Descriptive analyses were summarized by mean and standard deviation for continuous variables and absolute frequency and percentages for categorical variables. All statistical calculations were performed using StataCorp 2009 (Stata Statistical Software: Release 11. College Station, TX: StataCorp, LP).

## 3. Results

Clinical and demographic characteristics of the patients included in the study are shown in Table 1. Records were retrieved from a total of 255,499 patients with T2DM who during 2013 were prescribed antidiabetic pharmacological treatment based on a NIAD alone or in combination. The mean age of the patients was 68.0 years (standard deviation (SD) = 11.0), with a mean duration of T2DM of 8.0 years (SD = 5.6). The most common NIADs prescribed in pharmacologically treated cases were metformin (in 88.4% of patients), sulfonylureas (31.1%), and DPP4i (15.5%).

The clinical and demographic characteristics of the treated patients stratified by the pharmacological class of the glucose-lowering agent prescribed (alone or in combination) are shown in Table 2. In general, patients on metformin had a shorter T2DM duration, lower glycemic levels, a lower prevalence of renal failure, and fewer diabetic complications than patients treated with other NAIDs. Conversely, patients on insulin in combination with a NAID were at the other side of the spectrum and had the longest duration of the disease, highest HbA1c levels, and the highest rate of all diabetic complications.

The clinical characteristics of patients with contraindications and improper use of a NIAD are shown in Supplementary Table  1, in Supplementary Material available online at http://dx.doi.org/10.1155/2016/7502489. Patients with a contraindication were older and had a longer T2DM duration than those without a contraindication, but they did show lower HbA1c values and hence a better glycemic control.

### 3.1. Prescribing Patterns of NIADs in Patients with Contraindicated Comorbid Conditions

The number of T2DM patients with major contraindications and NIADs prescribed alone or in combination is shown in Table 3.

#### 3.1.1. Renal Failure

**Figure 1:**
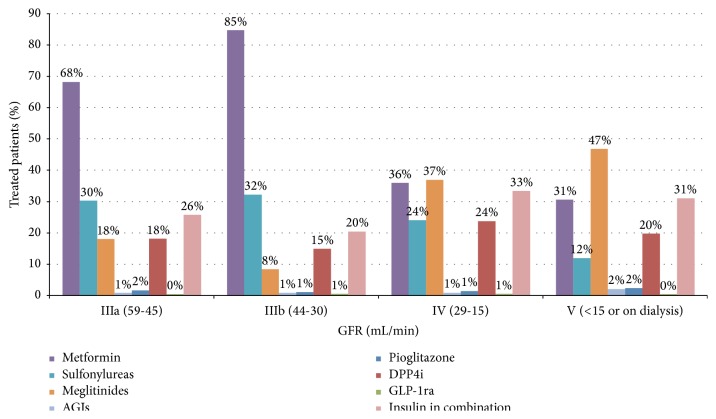
NIADs prescribed (alone or in combination) stratified by disease stage in patients with some degree of renal failure (GFR < 60 mL/min) (percentages are calculated for the total number of patients in each stage). AGI: alpha-glucosidase inhibitors; GLP-1ra: glucagon-like peptide-1 (GLP-1) receptor agonists; GFR: glomerular filtration rate; DPP4i: dipeptidyl peptidase-4 (DPP-4) inhibitors; NIAD: noninsulin antidiabetic drugs.

A total of 40,666 patients (20.1%) had some degree of renal failure (GFR < 60 mL/min). In 78% of these cases patients were treated with metformin, and 31.2% were treated with sulfonylureas, both of them theoretically contraindicated, while other agents were prescribed in less than 16% of cases. We observed that, even in cases of severe renal failure (GFR < 30 mL/min; *n* = 2,014; 1% of all treated patients), when they are formally contraindicated, a significant proportion of these patients were still on metformin or sulfonylureas (35.3% and 22.5%, resp.). Based on the degree of renal impairment (Figure 1), 36% of cases in stage IV and 31% in stage V were taking metformin, and 24% and 12% of cases in stages IV and V were taking sulfonylureas, respectively. However, in both stages IV and V the most frequently prescribed agents were meglitinides (37% and 47%, resp.). Moreover, we also observed that 1% of patients with severe renal failure were taking AGIs, and 0.4% were taking GLP-1ra.

#### 3.1.2. Heart Failure

A total of 13,276 patients (5.2%) had some degree of heart failure (Table 3). Again, metformin was the most frequently prescribed NIAD, and even in cases of moderate (class III) and severe (class IV) functional stages (a total of 1,222 patients), where metformin is formally contraindicated, it was prescribed in more than 60% of cases (67.8% in class III and 60.6% in class IV). Conversely, only 6 out of the 1,222 patients were on pioglitazone, which is also contraindicated in these 2 functional stages. In the less severe functional stages (classes I and II) only pioglitazone is contraindicated but was still prescribed in 5 out of the 938 patients in class I (0.5%) and in 14 out of the 2,300 patients in class II (0.6%) functional stage.

#### 3.1.3. Liver Dysfunction

A total of 1,447 patients had elevated liver enzymes (liver dysfunction; Table 3). In these cases, the vast majority of patients were on metformin or sulfonylureas (78.8% and 27.8%, resp.), which are not necessarily contraindicated except in cases of advanced liver failure. However, a small proportion of patients (0.5%) were prescribed pioglitazone, which, based on the SmPC, is contraindicated in patients with baseline GPT levels >2.5 times the upper limit of normal.

#### 3.1.4. Bladder Cancer

A history of bladder cancer was recorded for 3,073 patients (Table 3). Although the vast majority of these patients where on metformin or sulfonylureas, 33 of them (1.1%) were treated with pioglitazone, which is currently a formal contraindication in this condition.

## 4. Discussion

In the present study we identified a relatively high proportion of patients with T2DM and a comorbid disease with NIADs that are contraindicated or not recommended in cases of renal failure, heart failure, liver dysfunction, or history of bladder cancer.

Patients with a contraindication inappropriately taking a particular NIAD were older and had longer diabetes duration but had better glycemic control than patients without the same contraindication. This suggests that in spite of these patients being at risk of severe adverse events (e.g., hypoglycemia or lactic acidosis), treatment discontinuation could lead to a worsening of glycemic control, thus requiring a careful evaluation of the most appropriate NIADs to use in terms of efficacy and safety.

From our results, metformin, which is widely used as the initial pharmacological therapy for glycemic control in T2DM, was the NIAD that accounted for the vast majority of potentially inappropriate prescribing in patients with a comorbid disease: it was used in 35.3% of patients with severe renal failure (GFR < 30 mL/min) and in more than 60% of patients with moderate or severe heart failure. Because metformin has been associated with a risk of lactic acidosis, labeling contraindications include these conditions [10, 12, 23], but studies that have evaluated its prescribing pattern outside clinical recommendations reveal that it is actually used in a high proportion of cases in which major contraindications exist and in percentages similar to the figures that we observed [14–17]. A cross-sectional study conducted in Germany found that 73% of outpatients who were prescribed metformin had at least 1 contraindication, risk factors, or intercurrent illnesses necessitating its discontinuation [14]. A retrospective population-based study conducted in Scotland found that in 24.5% of patients who received metformin it was prescribed in spite of the presence of contraindications, and only 17.5% and 25% stopped metformin after admission with acute myocardial infarction and development of renal impairment, respectively [15]. A retrospective chart review of outpatients in the US found that about 25% of patients with 1 or more absolute contraindications (congestive heart failure or renal insufficiency) were prescribed metformin [17]. Finally, a retrospective study in Italy found that 60% of patients with 1 absolute contraindication or precautionary condition were on metformin at hospital admission, and in 41% of cases with 1 absolute contraindication it was not appropriately discontinued [16].

In the particular case of the use of metformin in patients with kidney disease, it has been consistently shown that prescribing restrictions, which recommend avoiding its use in patients with mild or moderate chronic kidney disease, are not actually followed in real-world practice [24]. Moreover, the incidence of lactic acidosis among patients on metformin is very low in stable mild-to-moderate renal dysfunction and not much different from the rates observed with other medications or the baseline incidence observed in T2DM [13, 24–26]. This has triggered some clinical guidelines to relax the cut-off, with current recommendations to stop metformin only when eGFR falls to <30 mL/min, but to reduce the dosing or use it with caution when eGFR values are between 45 and 30 mL/min [10, 12, 27, 28]. However, if we consider an eGFR <30 mL/min as the absolute contraindication, we still observed that 35.3% of patients in this stage were prescribed metformin in our setting, which is strikingly high.

The same trend has been observed with the use of metformin in patients with heart failure, with recent systematic reviews and meta-analyses showing that it is a safe option compared to other NIADs regarding the risk of heart failure or lactic acidosis [7, 29, 30]. Indeed, based on clinical evidence and these results, regulatory bodies in the US (Food and Drug Administration) in 2006 and in Canada (Health Canada) in 2010 removed the absolute contraindication of metformin in heart failure from the prescribing information and replaced it with a black box warning for its use in this population [31]. However, prescribing information has not been reviewed or modified in Europe, although the recent guideline of the European Society of Cardiology recognizes that they are widely and safely used in patients with heart failure and only recommends not to use it in case of severe renal failure or hepatic impairment [32]. Therefore, it seems reasonable to use metformin when the patient is stable and to avoid it in case of further impairment or hospitalization, as recommended by the recent American Diabetes Association guideline [10]. In our study, the rates of prescription of metformin in patients with III and IV heart failure functional stages were 67.8% and 60.6%, respectively. Moreover, we found that pioglitazone, which is also contraindicated in moderate or severe heart disease, was still prescribed to these patients (0.4% and 1% of patients with classes III and IV, resp.). In addition, the rates of inappropriate prescription of pioglitazone to patients for whom it is contraindicated in our study were <1.1% across different comorbidities (e.g., bladder cancer or liver dysfunction), which is much lower than the rate previously reported in a study conducted in Taiwan, which found that thiazolidinediones were inappropriately prescribed in about 10% of patients [33]. The reasons for this discrepancy are unclear, but the authors postulated that this high rate was due to the quick penetration of this drug class in the local market, while our low percentage could in turn be linked to a comparatively low penetrance in the Spanish market.

To our knowledge there are no studies assessing the rates of inappropriate prescription of sulfonylureas in formal contraindications, but many members of this class are associated with an increased risk of hypoglycemic episodes in patients with renal impairment or chronic liver disease [13]. We found figures that may be considered relatively high, as they were prescribed to 22.5% of patients with GFR <30 mL/min. In addition, sulfonylureas were prescribed in 27.8% of patients with liver dysfunction and metformin in 78.8% of such cases. However, they are only formally contraindicated in cases of advanced liver failure. Since we defined liver dysfunction as an elevation of liver enzymes >3 times the upper normal limits and this can be observed across different liver conditions, these do not actually correspond to real inappropriate prescriptions except in the case of pioglitazone (used in 0.5% of patients with liver dysfunction), which is specifically contraindicated when liver enzymes are elevated.

The present study has advantages and limitations that must be acknowledged. The main advantage is that we used a primary care database with high quality records that reflects real-life clinical practices in a large population of T2DM treated patients. However, and inherent to most retrospective studies, some of the studied variables were not always properly recorded in the medical records; for instance, there were 22% of patients without data on HbA1c values, 33% without data on GFR, or 66% for whom the NYHA functional class was not registered. Moreover, we cannot rule out a poor registration of comorbidities or at-risk conditions that could have underestimated the results. In addition, the retrospective design precludes determining whether clinicians were actually aware that they were prescribing against the label or clinical guidelines recommendations or the drugs were given in spite of the contraindication based on weighted individual risk-benefits. For instance, it is probable that the high rates of inappropriate or potentially inappropriate prescription of metformin is partly due to the fact that the risk-benefit in patients with nonabsolute contraindications favors its use in terms of the associated reduction of the risk of diabetes related complications, in particular macrovascular diseases. Finally, we could not estimate in what proportion of cases the particular drug was discontinued after the contraindicated condition developed and the at-risk condition was thus prevented, and we could not quantify either the incidence of adverse reactions after an inappropriate prescription (e.g., lactic acidosis, severe hypoglycemia episodes, or heart failure) or whether the drug was discontinued in case of a drug-related adverse event.

In summary, our results show that some NIADs, and in particular metformin, are frequently used in patients at high risk of complications when they are contraindicated or not recommended by the accompanying prescribing information or clinical guidelines. Prescribing of antidiabetic drugs to unsuitable patients has clinical consequences associated with an increased risk of adverse reactions and suboptimal glycemic control, and it is also associated with an economic impact relative to patients treated according to guidelines [34–37]. However, current guidelines or expert consensus does not always give clear recommendations on the use of specific NIADs in T2DM patients with a comorbid disease, probably as a result of a lack of clinical trials enrolling high-risk subjects, which may in turn result in a lack of practical advice for physicians, facilitating potentially inappropriate prescribing [13].

## 5. Conclusions

There is a need to increase awareness of potential inappropriate prescribing and to monitor the quality of prescribing patterns in order to help physicians and policymakers to yield better clinical outcomes in T2DM. This could be accomplished through the implementation of security reminders in the electronic clinical records so that physicians are aware of an existing complication that would require dose adjustment, discontinuing, or not even starting on a particular drug. Moreover, specific educational programs aimed at reducing the failure to recognize contraindications in patients with comorbid conditions and improving knowledge on currently available pharmaceutical products would be of great benefit to improve the management of the disease.

## Supplementary Material

Supplementary Table 1 shows the clinical characteristics of patients based on the particular NIAD prescribed and the presence or absence of contraindications.

## Figures and Tables

**Table 1:** Demographic and clinical characteristics of T2DM patients included in the study.

Characteristic	*N* = 255,499
Age, mean (SD), years	68.0 (11.0)
Gender, *n* (%)	
Female	114,181 (44.7%)
Male	141,318 (55.3%)
T2DM duration, mean (SD), years	8.0 (5.6)
HbA1c, mean (SD), %^*∗*^	7.3 (1.4)
BMI, mean (SD), kg/m^2^	30.3 (5.2)
Renal failure (GFR < 60 mL/min), *n* (%)^†^	40,666 (20.1%)
Severe renal failure (GFR < 30 mL/min), *n* (%)^†^	2,014 (1.0%)
Complications, *n* (%)	
Patients with registered severe hypoglycemia episodes	463 (0.2%)
Diabetic retinopathy	19,857 (7.8%)
ACR > 300 mg/g	6,661 (2.6%)
Diabetic neuropathy	7,509 (2.9%)
Ischemic heart disease	31,145 (12.2%)
Stroke	15,158 (5.9%)
Peripheral artery disease	12,295 (4.8%)
Heart failure	13,276 (5.2%)
Any macrovascular complication	51,007 (20.0%)
Glucose-lowering treatment, alone or in combination, *n* (%)	
Metformin	225,753 (88.4%)
Sulfonylureas	79,472 (31.1%)
Meglitinides	16,941 (6.6%)
AGIs	1,877 (0.7%)
Pioglitazone	3,290 (1.3%)
DPP4i	39,682 (15.5%)
GLP-1ra	2,374 (0.9%)
Insulin with a NIAD	46,150 (18.1%)

^*∗*^Out of 199,523 patients with available HbA1c records.

^†^Out of 195,674 patients with available GFR records.

ACR: albumin/creatinin ratio; AGI: alpha-glucosidase inhibitors; BMI: body mass index; DPP4i: dipeptidyl peptidase-4 (DPP-4) inhibitors; GLP-1ra: glucagon-like peptide-1 (GLP-1) receptor agonists; GFR: glomerular filtration rate; HbA1c: glycated hemoglobin; NIAD: noninsulin antidiabetic drug; SD: standard deviation; T2DM: type 2 diabetes mellitus.

**Table 2:** Demographic and clinical characteristics of T2DM patients stratified by the pharmacological class of the glucose-lowering agents prescribed (alone or in combination).

Characteristic	Metformin	Sulfonylureas	Meglitinides	AGIs	Pioglitazone	DPP4i	GLP-1ra	Insulin with NIAD
	(*n* = 225,753)	(*n* = 79,742)	(*n* = 16,941)	(*n* = 1,877)	(*n* = 3,290)	(*n* = 39,682)	(*n* = 2,374)	(*n* = 46,150)
Age, mean (SD), years	67.7 (11.4)	69.2 (11.2)	71.6 (10.9)	74.4 (9.9)	67.1 (10.6)	67.6 (10.9)	59.5 (9.5)	68.6 (11.0)
Gender, *n* (%)								
Female	99,082 (43.9)	34,648 (43.6)	7,867 (46.4)	850 (45.3)	1,561 (47.4)	17,199 (43.3)	1,281 (54.0)	22,679 (49.1)
Male	126,671 (56.1)	44,824 (56.4)	9,074 (53.6)	1,027 (54.7)	1,729 (52.6)	22,483 (56.7)	1,093 (46.0)	23,471 (50.9)
T2DM duration, mean (SD), years	7.9 (5.6)	9.3 (5.3)	9.9 (5.8)	10.7 (5.6)	10.6 (5.6)	9 (5.4)	8.7 (5.0)	11.4 (6.5)
HbA1c, mean (SD), %^*∗*^	7.3 (1.3)	7.6 (1.4)	7.7 (1.4)	7.2 (1.3)	7.7 (1.4)	7.7 (1.4)	7.9 (1.6)	8.3 (1.6)
BMI, mean (SD), kg/m^2^	30.3 (5.1)	30.0 (5.1)	29.9 (5.1)	28.7 (5.0)	32.2 (5.7)	30.4 (5.2)	37.0 (6.0)	31.0 (5.5)
ACR, mean (SD), mg/g	39.8 (143.7)	42.3 (149.7)	71.6 (223.7)	34.2 (88.3)	50.1 (191.8)	50.1 (176.7)	45.5 (137.2)	71.2 (213.3)
Complications, *n* (%)								
Diabetic retinopathy	17,533 (7.8)	6,594 (8.3)	2,059 (12.2)	190 (10.1)	407 (12.4)	3,766 (9.5)	308 (13.0)	9,980 (21.6)
Diabetic nephropathy^†^/ACR > 300 mg/g	5,455 (2.4)	2,171 (2.7)	856 (5.1)	58 (3.1)	131 (4.0)	1,312 (3.3)	105 (4.4)	2,465 (5.3)
Diabetic neuropathy	6,545 (2.9)	2,313 (2.9)	755 (4.5)	62 (3.3)	150 (4.6)	1,399 (3.5)	137 (5.8)	3,683 (8.0)
Ischemic heart disease	26,433 (11.7)	9,818 (12.4)	2,842 (16.8)	263 (14.0)	274 (8.3)	5,177 (13.0)	291 (12.3)	8,094 (17.5)
Stroke	12,777 (5.7)	4,541 (5.7)	1,363 (8.0)	123 (6.6)	148 (4.5)	2,062 (5.2)	80 (3.4)	3,904 (8.5)
Peripheral artery disease	10,470 (4.6)	3,956 (5.0)	1,272 (7.5)	86 (4.6)	155 (4.7)	2,045 (5.2)	80 (3.4)	3,954 (8.6)
Heart failure	9,711 (4.3)	4,266 (5.4)	1,698 (10.0)	120 (6.4)	82 (2.5)	2,063 (5.2)	113 (4.8)	3,780 (8.2)
Any macrovascular complication	43,440 (19.2)	16,018 (20.2)	331 (2.0)	413 (22.0)	509 (15.5)	8,105 (20.4)	388 (16.3)	13,284 (28.8)

^*∗*^Out of 199,523 patients with available HbA1c records.

^†^Out of 195,674 patients with available GFR records.

ACR: albumin/creatinine ratio; AGI: alpha-glucosidase inhibitors; BMI: body mass index; DPP4i: dipeptidyl peptidase-4 (DPP-4) inhibitors; GLP-1ra: glucagon-like peptide-1 (GLP-1) receptor agonists; GFR: glomerular filtration rate; HbA1c: glycated haemoglobin; NIAD: noninsulin antidiabetic drug; SD: standard deviation; T2DM: type 2 diabetes mellitus.

**Table 3:** Number of T2DM patients (%) with relevant contraindications and glucose-lowering agents prescribed (alone or in combination). Percentages indicate the proportion of patients treated with each NAID with respect to the total number of patients with the condition.

Condition	Total patients with the condition	Metformin (*n* = 225,753)	Sulfonylureas (*n* = 79,742)	Meglitinides (*n* = 16,941)	AGIs (*n* = 1,877)	Pioglitazone (*n* = 3,290)	DPP4i (*n* = 39,682)	GLP-1ra (*n* = 2,374)
Renal failure^*∗*^ (GFR < 60 mL/min), *n* (%)	40,666 (20.1)	31,727 (78.0)	12,695 (31.2)	5,019 (12.3)	381 (0.9)	514 (1.3)	6,559 (16.1)	198 (0.5)

Severe renal failure^*∗*^ (GFR < 30 mL/min), *n* (%)	2,014 (1.0)	711 (35.3)	545 (22.5)	769 (38.1)	21 (1.0)	31 (1.5)	468 (23.2)	9 (0.4)

Heart failure, *n* (%)	13,276 (5.2)	9,711 (73.1)	4,266 (32.1)	1,698 (12.8)	120 (0.9)	82 (0.6)	2,063 (15.5)	113 (0.8)

Heart failure (NYHA functional stage)^†^, *n* (%)								
Class I	938	761 (81.1)	285 (30.4)	88 (9.4)	6 (0.6)	5 (0.5)	161 (17.2)	6 (0.6)
Class II	2,300	1,720 (74.8)	705 (30.7)	283 (12.3)	21 (0.9)	14 (0.6)	353 (15.3)	30 (1.3)
Class III	1,118	758 (67.8)	321 (28.7)	197 (17.6)	9 (0.8)	5 (0.4)	163 (14.6)	11 (1.0)
Class IV	104	63 (60.6)	28 (26.9)	18 (17.3)	2 (1.9)	1 (1.0)	16 (15.4)	1 (1.0)

Liver dysfunction, *n* (%)	1,447 (0.6)	1,140 (78.8)	402 (27.8)	114 (7.9)	12 (0.8)	7 (0.5)	221 (15.3)	14 (1.0)

Bladder cancer, *n* (%)	3,073 (1.2)	2,573 (83.7)	953 (31.0)	277 (9.0)	23 (0.7)	33 (1.1)	484 (15.8)	11 (0.4)

^**∗**^Out of 195,674 patients with available GFR records.

^†^Out of 4,458 patients with available New York Heart Association (NYHA) functional classification records.

AGI: alpha-glucosidase inhibitors; DPP4i: dipeptidyl peptidase-4 (DPP-4) inhibitors; GLP-1ra: glucagon-like peptide-1 (GLP-1) receptor agonists; GFR: glomerular filtration rate.
