# Supplementary material for: Noninsulin Antidiabetic Drugs for Patients with Type 2 Diabetes Mellitus: Are We Respecting Their Contraindications?
Source: J Diabetes Res. 2016 Jan 6;2016:7502489. doi: 10.1155/2016/7502489 (PMC4736814; doi:10.1155/2016/7502489)
Supplement: Supplementary file 1 — Supplementary Table 1 shows the clinical characteristics of patients based on the particular NIAD prescribed and the presence or absence of contraindications. [file 7502489.f1.docx]

**Supplementary Table 1.** Clinical characteristics of patients based on the particular NIAD prescribed and the presence or absence of contraindications.^a^

|  | **Treated with Metformin** | | **Treated with Sulfonylureas** | | **Treated with AGIs** | | **Treated with Pioglitazone** | | **Treated with GLP-1rA** | |
| --- | --- | --- | --- | --- | --- | --- | --- | --- | --- | --- |
|  | *Without contraindications* | *Any contraindication* | *Without contraindication* | *Any contraindication* | *Without contraindications* | *Any contraindication* | *Without contraindications* | *Any contraindication* | *Without contraindications* | *Any contraindication* |
| N | 171535 | 2742 | 60642 | 933 | 1348 | 25 | 2295 | 65 | 1547 | 7 |
| Age | 68.0 ±11.2 | 73,9 ±10,5 *** | 69.3 ±11.0 | 75.0 ±10.8 *** | 74.6 ±9.53 | 80.7 ±7.87 ** | 67.2 ±10.7 | 73.1 ±9.08 *** | 59.6 ±9.52 | 70.7 ±4.75 ** |
| T2DM duration | 7.97 ±5.61 | 9.43 ±5.98 *** | 9. 39 ±5.34 | 10.2 ±5.63 *** | 11.1 ±5.88 | 10.0 ±4.24 | 10.8 ±5.68 | 10.5 ±5.31 | 8.78 ±5.11 | 9.92 ±3.31 |
| HbA1c | 7.33 ±1.35 | 7.25 ±1.44 ** | 7.63 ±1.38 | 7.30 ±1.46 *** | 7.20 ±1.26 | 6.90 ±1.03 | 7.67 ±1.41 | 7.11 ±0.87 ** | 7.97 ±1.58 | 7.27 ±2.31 |
| BMI | 30.3 ±5.09 | 30.7 ±5.80 *** | 30.0 ±5.05 | 29.9 ±5.29 | 28.6 ±4.90 | 29.4 ±4.29 | 32.2 ±5.63 | 32.6 ±6.11 | 37.3 ±5.95 | 32.8 ±1.53 |
| Heart Failure (any NYHA class) | 6816 (4.0) | 1062 (38.7) *** | 3234 (5.3) | 169 (18.1) *** | 88 (6.5) | 3 (12) | 35 (1.5) | 28 (43.1) *** | 73 (4.7) | 1 (14.3) |
| Liver dysfunction | --- | 1140 (41.6) | --- | 402 (43.1) | 11 (0.8) | 0 (0) | --- | 7 (10.8) | 9 (0.6) | 0 (0) |
| Severe renal failure (GFR<30mL/min) | --- | 806 (36.4) | --- | 532 (66.1) | --- | 25 (100) | 33 (1.4) | 1 (1.9) | --- | 7 (100) |

Numbers in parenthesis indicate the percentage of the corresponding column

* p<0.05

** p<0.01

*** p<0.001

^a^ Major contraindications for each drug class:

- Metformin: Glomerular Filtrate Rate (GFR) <30 mL/min; heart failure class III and IV New York Heart Association (NYHA); liver dysfunction
- Sulfonylureas: GFR <30 mL/min; liver dysfunction
- Pioglitazone: heart failure class I to IV NYHA; bladder cancer; liver dysfunction
- GLP-1ra: GFR<30 mL/min
- AGI: GFR<30 mL/min; liver dysfunction
